# Effects of Noise on the Behavioral and Neural Categorization of Speech

**DOI:** 10.3389/fnins.2020.00153

**Published:** 2020-02-27

**Authors:** Gavin M. Bidelman, Lauren C. Bush, Alex M. Boudreaux

**Affiliations:** ^1^Institute for Intelligent Systems, University of Memphis, Memphis, TN, United States; ^2^School of Communication Sciences and Disorders, University of Memphis, Memphis, TN, United States; ^3^Department of Anatomy and Neurobiology, University of Tennessee Health Sciences Center, Memphis, TN, United States

**Keywords:** auditory event-related potentials (ERPs), categorical perception, speech-in-noise (SIN) perception, cocktail party effect, EEG

## Abstract

We investigated whether the categorical perception (CP) of speech might also provide a mechanism that aids its perception in noise. We varied signal-to-noise ratio (SNR) [clear, 0 dB, −5 dB] while listeners classified an acoustic-phonetic continuum (/u/ to /a/). Noise-related changes in behavioral categorization were only observed at the lowest SNR. Event-related brain potentials (ERPs) differentiated category vs. category-ambiguous speech by the P2 wave (~180–320 ms). Paralleling behavior, neural responses to speech with clear phonetic status (i.e., continuum endpoints) were robust to noise down to −5 dB SNR, whereas responses to ambiguous tokens declined with decreasing SNR. Results demonstrate that phonetic speech representations are more resistant to degradation than corresponding acoustic representations. Findings suggest the mere process of binning speech sounds into categories provides a robust mechanism to aid figure-ground speech perception by fortifying abstract categories from the acoustic signal and making the speech code more resistant to external interferences.

## Introduction

A basic tenet of perceptual organization is that sensory phenomena are subject to invariance: similar features are mapped to common identities (equivalence classes) by assigning similar objects to the same membership (Goldstone and Hendrickson, 2010), a process known as categorical perception (CP). In the context of speech, CP is demonstrated when gradually morphed sounds along an equidistant acoustic continuum are heard as only a few discrete classes (Liberman et al., 1967; Pisoni, 1973; Harnad, 1987; Pisoni and Luce, 1987; Bidelman et al., 2013). Equal physical steps along a signal dimension do not produce equivalent changes in percept (Holt and Lotto, 2006). Rather, listeners treat sounds within a given category as perceptually similar despite their otherwise dissimilar acoustics. Skilled categorization is particularly important for spoken and written language, as evidenced by its role in reading acquisition (Werker and Tees, 1987; Mody et al., 1997), sound-to-meaning learning (Myers and Swan, 2012; Reetzke et al., 2018), and putative deficits in language-based learning disorders (e.g., specific language impairment, dyslexia; Werker and Tees, 1987; Noordenbos and Serniclaes, 2015; Calcus et al., 2016). To arrive at categorical decisions, acoustic cues are presumably weighted and compared against internalized “templates” in the brain, built through repetitive exposure to one’s native language (Kuhl, 1991; Iverson et al., 2003; Guenther et al., 2004; Bidelman and Lee, 2015).^1^

**FIGURE 1:**
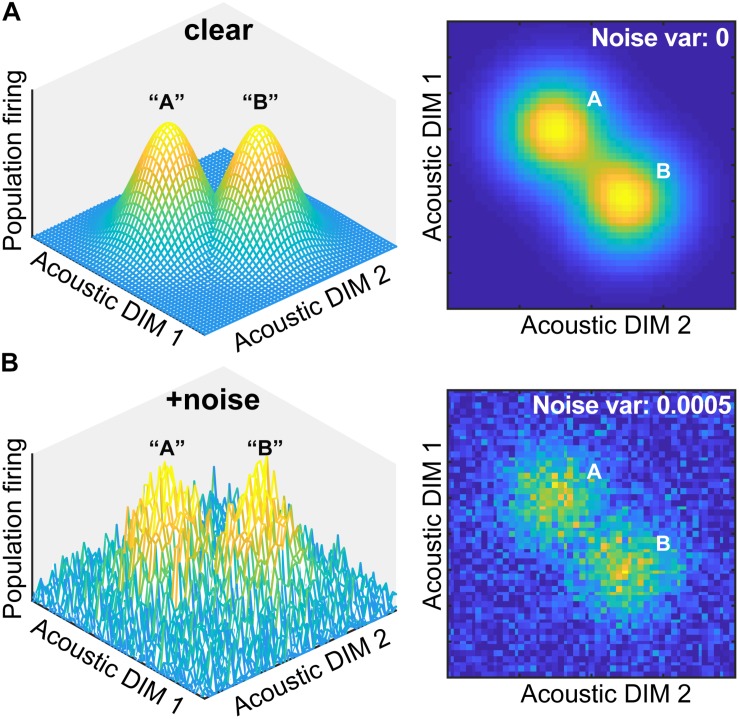
Theoretical framework for noise-related influences on categorical speech representations. **(A)** The neural representation of speech is modeled as a multidimensional feature space where populations of auditory cortical neurons code different dimensions (DIM) of the input. DIMS here are arbitrary but could reflect any behaviorally relevant feature of speech (e.g., F0, duration, etc.) Both 3D and 2D representations are depicted here for two stimulus classes. Categorical coding (modeled as a Gaussian mixture) is reflected by an increase in local firing rate for perceptually similar stimuli (“A” and “B”). **(B)** Noise blurs physical acoustic details yet spares categories as evidenced by the resilience of the peaks in neural space. Neural noise was modeled by changing the variance of additive Gaussian white noise.

Beyond providing observers a smaller, more manageable perceptual space, why else might the perceptual-cognitive system build equivalence classes? Goldstone and Hendrickson (2010) argue that one reason is that categories “are relatively imperious to superficial similarities. Once one has formed a concept that treats [stimuli] as equivalent for some purposes, irrelevant variations among [stimuli] can be greatly deemphasized” (Goldstone and Hendrickson, 2010, p. 2). Based on this premise, we posited that categories might also aid degraded speech perception if phonetic categories are somehow more resistant to noise (Gifford et al., 2014; Helie, 2017). Indeed, categories (a higher-level code) are thought to be more robust to noise degradations than physical surface features of a signal (lower-level sensory code) (Helie, 2017; Bidelman et al., 2019). A theoretical example of how categorical processing might aid the perception of degraded speech is illustrated in Figure 1.

Consider the neural representation of speech as a multidimensional feature space. Populations of auditory cortical neurons code different dimensions of the acoustic input. Categorical coding could be reflected as an increase (or conversely, decrease) in local firing rate for stimuli that are perceptually similar despite their otherwise dissimilar acoustics (“A” and “B”) (e.g., Recanzone et al., 1993; Guenther and Gjaja, 1996; Guenther et al., 2004). Although noise interference would blur physical acoustic details and create a noisier cortical map, categories would be partially spared—indicated by the remaining “peakedness” in the neural space. Thus, both the construction of perceptual objects and natural discrete binning process of CP might enable category members to “pop out” among a noisy feature space (e.g., Nothdurft, 1991; Perez-Gay et al., 2018). Consequently, the mere process of grouping speech sounds into categories might aid comprehension of speech-in-noise (SIN)—assuming those representations are not too severely compromised and remain distinguishable from noise itself. This theoretical framework provides the basis for the current empirical study and is supported by recent behavioral data and modeling (Bidelman et al., 2019).

Building on our recent efforts to decipher the neurobiology of noise-degraded speech perception and physiological mechanisms supporting robust perception (for review, see Bidelman, 2017), this study aimed to test whether speech sounds carrying strong phonetic categories are more resilient to the deleterious effects of noise than categorically ambiguous speech sounds. When category-relevant dimensions are less distinct and perceptual boundaries are particularly noisy, additional mechanisms for enhancing separation must be engaged (Livingston et al., 1998). We hypothesized the phonetic groupings inherent to speech may be one such mechanism. The effects of noise on the auditory neural encoding of speech are well documented in that masking generally weakens and delays event-related brain potentials (ERPs) (e.g., Alain et al., 2012; Billings et al., 2013; Bidelman and Howell, 2016). However, because phonetic categories reflect a more abstract, higher-level representation of speech (i.e., acoustic + phonetic code), we reasoned they would be more robust to noise than physical features of speech that do not engage phonetic-level processing (i.e., acoustic code) (cf. Helie, 2017; Bidelman et al., 2019). To test this possibility, we recorded high-density ERPs while listeners categorized speech continua in different levels of acoustic noise. The critical comparison was between responses to stimuli at the endpoints vs. midpoint of the acoustic-phonetic continuum. Because noise should have a uniform effect on token comprehension (i.e., it is applied equally across the continuum), stronger changes at the mid- vs. endpoint of the continuum with decreasing signal-to-noise ratio (SNR) would indicate a differential impact of noise on category representations. We predicted that if the categorization process aids figure-ground perception, speech tokens having a clear phonetic identity (continuum endpoints) would elicit lesser noise-related change in the ERPs than phonetically ambiguous tokens (continuum midpoint), which have a bistable (ambiguous) percept and lack a clear phonetic identity.

## Materials and Methods

### Participants

Fifteen young adults (3 male, 12 females; age: *M* = 24.3, *SD* = 1.7 years) were recruited from the University of Memphis student body. Sample size was based on previous studies on categorization including those examining noise-related changes in CP (*n* = 9–17; Myers and Blumstein, 2008; Liebenthal et al., 2010; Bidelman et al., 2019). All exhibited normal hearing sensitivity confirmed via a threshold screening (i.e., <20 dB HL, audiometric frequencies 250 – 8000 Hz). Each participant was strongly right-handed (87.0 ± 18.2% laterality index; Oldfield, 1971) and had obtained a collegiate level of education (17.8 ± 1.9 years). Musical training is known to modulate categorical processing and SIN listening abilities (Parbery-Clark et al., 2009; Bidelman and Krishnan, 2010; Zendel and Alain, 2012; Bidelman et al., 2014; Bidelman and Alain, 2015b; Yoo and Bidelman, 2019). Consequently, we required that all participants had minimal music training throughout their lifetime (mean years of training: 1.3 ± 1.8 years). All were paid for their time and gave informed consent in compliance with the Declaration of Helsinki and a protocol approved by the Institutional Review Board at the University of Memphis.

### Speech Continuum and Behavioral Task

We used a synthetic five-step vowel continuum spanning from “u” to “a” to assess the neural correlates of CP (Bidelman et al., 2014; Bidelman and Alain, 2015b; Bidelman and Walker, 2017). Each token of the continuum was separated by equidistant steps acoustically based on first formant frequency (F1). Tokens were 100 ms, including 10 ms of rise/fall time to reduce spectral splatter in the stimuli. Each contained identical voice fundamental (F0), second (F2), and third formant (F3) frequencies (F0: 150, F2: 1090, and F3: 2350 Hz), chosen to roughly approximate productions from male speakers (Peterson and Barney, 1952). Natural speech (and vowels) can vary along multiple acoustic dimensions. However, auditory ERPs are also highly sensitive to multiple acoustic features. Thus, although our synthetic tokens are somewhat artificial, we chose to parametrize only one acoustic cue (F1) to avoid confounding the interpretation of our ERP effects. Consequently, F1 was parameterized over five equal steps between 430 and 730 Hz such that the resultant stimulus set spanned a perceptual phonetic continuum from /u/ to /a/ (Bidelman et al., 2013).^2^ Speech stimuli were delivered binaurally at 75 dB SPL through shielded insert earphones (ER-2; Etymotic Research) coupled to a TDT RP2 processor (Tucker Davis Technologies).

**FIGURE 2:**
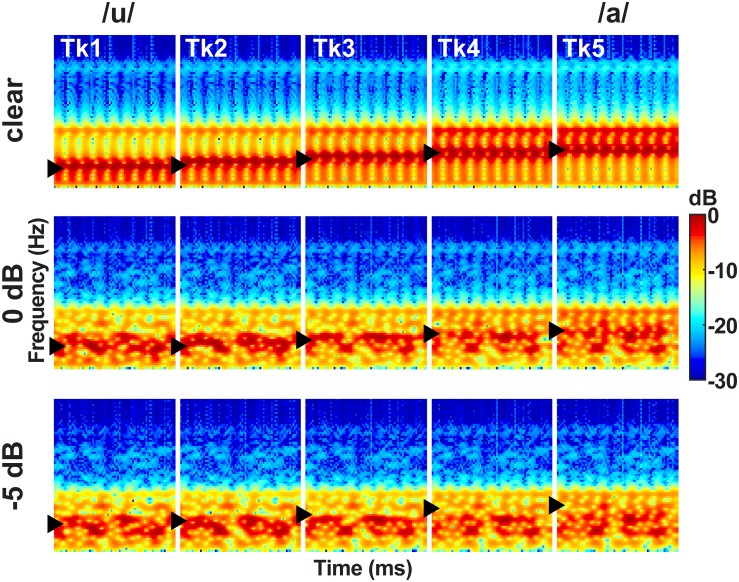
Acoustic spectrograms of the speech continuum as a function of SNR. Vowel first formant frequency was parameterized over five equal steps (430–730 Hz, ▶), resulting in a perceptual phonetic continuum from /u/ to /a/. Token durations were 100 ms. Speech stimuli were presented at 75 dB SPL with noise added parametrically to vary SNR.

This same speech continuum was presented in one of three noise blocks varying in SNR: clear, 0 dB SNR, −5 dB SNR (Figure 2). These noise levels were selected based on extensive pilot testing which confirmed they differentially hindered speech perception. The masker was a speech-shaped noise based on the long-term power spectrum (LTPS) of the vowel set. Pilot testing showed more complex forms of noise (e.g., multitasker babble) were too difficult for concomitant vowel identification, necessitating the use of simpler LTPS noise. Noise was presented continuously so it was not time-locked to the stimulus presentation, providing a constant backdrop of acoustic interference during the categorization task (e.g., Alain et al., 2012; Bidelman and Howell, 2016; Bidelman et al., 2018). SNR was manipulated by changing the level of the masker to ensure SNR was inversely correlated with overall sound level (Binder et al., 2004). Noise block order was randomized within and between participants.

The task was otherwise identical to our previous neuroimaging studies on CP (e.g., Bidelman et al., 2013; Bidelman and Alain, 2015b; Bidelman and Walker, 2017). During EEG recording, listeners heard 150 trials of each individual speech token (per noise block). On each trial, they were asked to label the sound with a binary response (“u” or “a”) as quickly and accurately as possible. Following listeners’ behavioral response, the interstimulus interval (ISI) was jittered randomly between 800 and 1000 ms (20 ms steps, uniform distribution) to avoid rhythmic entrainment of the EEG and the anticipation of subsequent stimuli.

Customarily, a pairwise (e.g., 1 vs. 2, 2 vs. 3, etc.) discrimination task complements identification functions in establishing CP (Pisoni, 1973). While discrimination is somewhat undesirable in the current study given the use of time-varying background noise (task-irrelevant noise cues may artificially inflate discrimination performance), we nevertheless measured 2-step paired discrimination in an additional sample (*n* = 7) of listeners to further validate our claims from the main identification experiment (see Supplementary Material).

### EEG Recording and Preprocessing

EEGs were recorded from 64 sintered Ag/AgCl electrodes at standard 10–10 scalp locations (Oostenveld and Praamstra, 2001). Continuous data were digitized using a sampling rate of 500 Hz (SynAmps RT amplifiers; Compumedics Neuroscan) and an online passband of DC-200 Hz. Electrodes placed on the outer canthi of the eyes and the superior and inferior orbit monitored ocular movements. Contact impedances were maintained <10 kΩ during data collection. During acquisition, electrodes were referenced to an additional sensor placed ∼1 cm posterior to the Cz channel.

EEG pre-processing was performed in BESA^®^ Research (v7) (BESA, GmbH). Ocular artifacts (saccades and blinks) were first corrected in the continuous EEG using a principal component analysis (PCA) (Picton et al., 2000). Cleaned EEGs were then filtered (1–30 Hz), epoched (−200 – 800 ms), baseline corrected to the pre-stimulus interval, and averaged in the time domain resulting in 15 ERP waveforms per participant (5 tokens * 3 noise conditions). For analysis, data were re-referenced using BESA’s reference-free virtual montage. This montage computes a spherical spline-interpolated voltage (Perrin et al., 1989) for each channel relative to the mean voltage over 642 equidistant locations covering the entire sphere of the head. This montage is akin to common average referencing but results in a closer approximation to true reference free waveforms (Scherg et al., 2002). However, results were similar using a common average reference (data not shown).

ERP quantification focused on the latency range following the P2 wave as previous studies have shown the neural correlates of CP emerge around the timeframe of this component (Bidelman et al., 2013; Bidelman and Alain, 2015b; Bidelman and Lee, 2015; Bidelman and Walker, 2017, 2019). Guided by visual inspection of grand averaged data, it was apparent that P2 was not well defined as a single isolated wave, rather, it occurred in a complex. Thus, we measured the amplitude of the evoked potentials as the positive-going deflection between 180–320 ms. This window covered what are likely the P2 and following P3b-like deflections. To evaluate whether ERPs showed category-related effects, we averaged response amplitudes to endpoint tokens at the endpoints of the continuum and compared this combination to the ambiguous token at its midpoint (e.g., Liebenthal et al., 2010; Bidelman, 2015; Bidelman and Walker, 2017; Bidelman and Walker, 2019). This contrast [i.e., mean(Tk1, Tk5) vs. Tk3] allowed us to assess the degree to which neural responses reflected “category level-effects” (Toscano et al., 2018) or “phonemic categorization” (Liebenthal et al., 2010). The rationale for this analysis is that it effectively minimizes stimulus-related differences in the ERPs, thereby isolating categorical/perceptual processing. For example, Tk1 and Tk5 are expected to produce distinct ERPs due to exogenous acoustic processing alone. However, comparing the average of these responses (i.e., mean[Tk1, Tk5]) to that of Tk3 allowed us to better isolate ERP modulations related to the process of categorization (Liebenthal et al., 2010; Bidelman and Walker, 2017, 2019).^3^

Averaging endpoint responses doubles the number of trials for the endpoint tokens relative to the ambiguous condition, which could mean differences were attributable to SNR of the ERPs rather than CP effects, *per se* (Hu et al., 2010). To rule out this possibility, we measured the SNR of the ERPs as 10log(RMS_ERP_/RMS_*baseline*_) (Bidelman, 2018) where RMS_ERP_ and RMS_baseline_ were the RMS amplitudes of the ERP (signal) portion of the epoch window (0–800 ms) and pre-response baseline period (−200 – 0 ms ms), respectively. Critically, SNR of the ERPs did not differ across conditions (*F*_5_,_70_ = 0.56, *p* = 0.73), indicating that neural activity was not inherently noisier for a given token type or acoustic noise level. Additionally, a split-half analysis (even vs. odd trials) indicated excellent reliability of ERP amplitudes at each SNR condition (Cronbach’s-α*_*clean*_* = 0.94; α*_0 *dB*_* = 0.83; α*_–5 *dB*_* = 0.81) (Streiner, 2003), suggesting highly stable EEG responses within our sample, even in the noisiest listening conditions.

### Behavioral Data Analysis

Identification scores were fit with a sigmoid function *P* = 1/[1 + *e*^–^*^β1(*x*–β0)^*], where *P* is the proportion of trials identified as a given vowel, *x* is the step number along the stimulus continuum, and β*_0_* and β*_1_* the location and slope of the logistic fit estimated using non-linear least-squares regression. Comparing parameters between SNR conditions revealed possible differences in the location and “steepness” (i.e., rate of change) of the categorical boundary as a function of noise degradation. Larger β*_1_* values reflect steeper psychometric functions and thus stronger CP.

Behavioral speech labeling speeds (i.e., reaction times [RTs]) were computed as listeners’ median response latency across trials for a given condition. RTs outside 250–2500 ms were deemed outliers (e.g., fast guesses, lapses of attention) and were excluded from the analysis (Bidelman et al., 2013; Bidelman and Walker, 2017).

### Statistical Analysis

Unless otherwise noted, dependent measures were analyzed using a one-way, mixed model ANOVA (subject = random factor) with fixed effects of SNR (3 levels: clear, 0 dB, −5 dB) and token [5 levels: Tk1-5] (PROC GLIMMIX, SAS^®^ 9.4; SAS Institute, Inc.). Tukey–Kramer adjustments controlled Type I error inflation for multiple comparisons. The α-level for significance was *p* = 0.05. We used repeated measures correlations (rmCorr) (Bakdash and Marusich, 2017) to assess brain-behavior associations within each listener. Unlike conventional correlations, rmCorr accounts for non-independence among observations, adjusts for between subject variability, and measures within-subject correlations by evaluating the common intra-individual association between two measures. We used the *rmCorr* package (Bakdash and Marusich, 2017) in the R software environment (R Core Team, 2018).

## Results

### Behavioral Identification (%, RTs)

**FIGURE 3:**
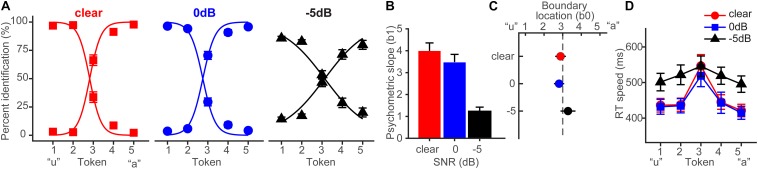
Behavioral speech categorization is robust to noise interference. **(A)** Perceptual psychometric functions for clear and degraded speech identification. Curves show an abrupt shift in perception when classifying speech indicative of discrete perception (i.e., CP). **(B)** Slopes and **(C)** locations of the perceptual boundary show speech categorization is robust even down to 0 dB SNR. **(D)** Speech classification speeds (RTs) show a categorical pattern for clear and 0 dB SNR speech; participants are slower at labeling ambiguous tokens (midpoint) relative to those with a clear phonetic label (endpoints) (Pisoni and Tash, 1974; Bidelman and Walker, 2017). A categorical RT effect is not observed for highly degraded speech (–5 dB SNR). errorbars = ± s.e.m. Figure adapted from Lewis and Bidelman (2020).

Behavioral identification functions are shown across the different noise SNRs in Figure 3A. Listeners’ identification was more categorical (i.e., dichotomous) for clear speech and became more continuous with poorer SNR. Analysis of the slopes (β*_1_*) confirmed a main effect of SNR (*F_2_,_28_* = 35.25, *p* < 0.0001) (Figure 3B). Tukey–Kramer contrasts revealed psychometric slopes were unaltered for 0 dB SNR relative to clear speech (*p* = 0.33). However, −5 dB SNR noise weakened categorization, flattening the psychometric function (−5 dB vs. 0 dB, *p* < 0.0001). These findings indicate the strength of categorical representations is resistant to acoustic interference. That is, even when signal and noise compete at equivalent levels, categorical processing persists. CP is weakened only for severely degraded speech (i.e., negative SNRs) where the noise exceeds the target signal.

Noise-related changes in the psychometric function could be related to uncertainty in category distributions (prior probabilities) (Gifford et al., 2014) or lapses of attention due to task difficulty rather than a weakening of speech categories, *per se* (Bidelman et al., 2019). To rule out this latter possibility, we used Bayesian inference (psignifit toolbox; Schütt et al., 2016) to estimate individual lapse (λ) and guess (γ) rates from participants’ identification data. Lapse rate (λ) was computed as the difference between the upper asymptote of the psychometric function and 100%, reflecting the probability of an “incorrect” response at infinitely high stimulus levels (i.e., responding “u” for Tk5; see Figure 3A). Guess rate (γ) was defined as the difference between the lower asymptote and 0. For an ideal observer λ = 0 and γ = 0. We found neither lapse (*F*_2_,_28_ = 2.41, *p* = 0.11) nor guess rate (*F*_2_,_28_ = 1.45, *p* = 0.25) were modulated by SNR. This helps confirm that while (severe) noise weakened CP for speech (Figure 3B), those effects were not driven by a lack of task vigilance or guessing, *per se* (Schütt et al., 2016; Bidelman et al., 2019).

The location of the perceptual boundary (Figure 3C) varied marginally with SNR but the shift was significant (*F*_2_,_28_ = 5.62, *p* = 0.0089). Relative to the clear condition, −5 dB SNR speech shifted the perceptual boundary rightward (*p* = 0.011). This indicates a small but measurable bias to report “u” (i.e., more frequent Tk1-2 responses) in the noisiest listening condition.^4^

Behavioral RTs, reflecting the speed of categorization, are shown in Figure 3D. An ANOVA revealed RTs were modulated by both SNR (*F*_2_,_200_ = 11.90, *p* < 0.0001) and token (*F*_4_,_200_ = 5.36, *p* = 0.0004). RTs were similar when classifying clear and 0 dB SNR speech (*p* = 1.0) but slowed in the −5 dB condition (*p* < 0.0001). Notably, *a priori* contrasts revealed this noise-related slowing in RTs was most prominent at the phonetic endpoints of the continuum (Tk1-2 and Tk4-5); at the ambiguous Tk3, RTs were identical across SNRs (*p*s > 0.69). This suggests that the observed RT effects in noise are probably not due to a general slowing of decision speed (e.g., attentional lapses) across the board but rather, are restricted to accessing categorical representations.

CP is also characterized by a slowing in RTs near the ambiguous midpoint of the continuum (Pisoni and Tash, 1974; Poeppel et al., 2004; Bidelman et al., 2013, 2014; Bidelman and Walker, 2017; Reetzke et al., 2018). Planned contrasts revealed this characteristic slowing in RTs for the clear [mean(Tk1,2,4,5) vs. Tk3; *p* = 0.0003] and 0 dB SNR (*p* = 0.0061) conditions. This categorical RT pattern was not observed at −5 dB SNR (*p* = 0.59). Collectively, our behavioral results suggest noise weakened the strength of CP in both the quality and speed of categorical decisions but only when speech was severely degraded. Perceptual access to categories was otherwise unaffected by low-level noise (i.e., ≥0 dB SNR).

Discrimination performance was uniformly high across vowel pairs and noise levels (mean = 83%; Supplementary Figure S2). However, this effect might be expected for vowel stimuli since listeners can exploit acoustic in addition to phonetic (categorical) cues (Pisoni, 1973). Nevertheless, “peaked discrimination” was apparent in the highest noise condition, indicative of categorical processing (see Supplementary Material).

### Electrophysiological Data

**FIGURE 4:**
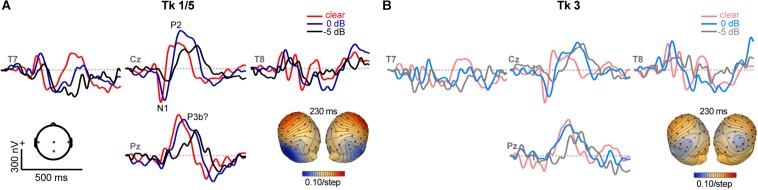
ERPs as a function of speech token and noise (SNR). Representative electrodes at central (Cz), temporal (T7/8) and parietal (Pz) scalp sites. Stimulus and noise-related modulations are most prominent at P2 and following (180–320 ms). **(A)** Phonetic speech tokens (Tk1, Tk5) elicit stronger ERPs than **(B)** ambiguous sounds without a clear category (Tk3). Noise weakens and prolongs the neural encoding of speech. Inserts show topographic maps at 230 ms. Hot/cool colors = positive/negative voltage.

**FIGURE 5:**
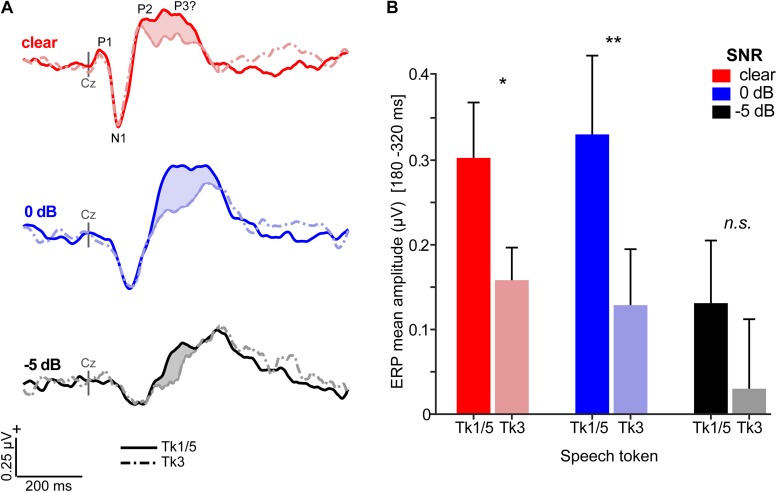
Categorical neural organization limits the degradative effects of noise on cortical speech processing. **(A)** Scalp auditory ERP waveforms (Cz electrode). Stronger responses are observed for phonetic exemplar vs. ambiguous speech tokens [i.e., mean(Tk1, Tk5) > Tk3; *shaded regions*] but this effect varies with SNR. **(B)** Mean ERP amplitude (180–320 ms window) is modulated by SNR and phonetic status. Categorical neural encoding (Tk1/5 > Tk3) is observed for all but the noisiest listening condition. errorbars = ± s.e.m. *p < 0.05; ** *p* < 0.01.

Grand average ERPs are shown across tokens and SNRs in Figures 4, 5 and Supplementary Figure S1. Predictably, noise delayed the ERP waves (Supplementary Figure S1), consistent with well-known masking effects and desynchronization in neural responses with acoustic interference (e.g., Alain et al., 2012; Billings et al., 2013; Ponjavic-Conte et al., 2013; Alain et al., 2014; Bidelman and Howell, 2016). Amplitude and latency analysis of the N1 revealed it was strongly modulated by SNR (N1_amp_: *F*_2_,_196_ = 18.95, *p* < 0.0001; N1_lat_: *F*_2_,_196_ = 114.74, *p* < 0.0001) but not token (N1_amp_: *F*_4_,_196_ = 0.27, *p* = 0.89; N1_lat_: *F*_4_,_196_ = 0.78, *p* = 0.54), consistent with previous ERP studies which have observed masking (Alain et al., 2014; Bidelman and Howell, 2016) but not categorical coding effects at N1 (Toscano et al., 2010; Bidelman et al., 2013) (Supplementary Figure S1). Instead, SNR- and token-related modulations were apparent starting around the P2 wave (∼180 ms) that persisted for another 200 ms. Visual inspection of the data indicated these modulations were most prominent at centro-parietal scalp locations. The enhanced positivity at these electrode sites following the auditory P2 might partly reflect differences in P3b amplitude (Alain et al., 2001). To quantify these effects, we measured the mean amplitudes in the 180–320 ms time window at the vertex channel (Cz) (Figure 5). To assess the degree to which ERPs showed categorical-level coding, we then pooled tokens Tk1 and Tk5 (those with clear phonetic identities) and compared these responses to the ambiguous Tk3 at the midpoint of the continuum (Bidelman, 2015; Bidelman and Walker, 2017). An ANOVA conducted on ERP amplitudes showed responses were strongly modulated by SNR (*F*_2_,_70_ = 8.54, *p* = 0.0005) and whether not the stimulus carried a strong phonetic label (Tk1/5 vs. Tk3: *F*_1_,_70_ = 19.11, *p* < 0.0001) (Figure 5B). The token x SNR interaction was not significant (*F*_2_,_70_ = 0.73, *p* = 0.49). However, planned contrasts by SNR revealed that neural activity differentiated phonetically unambiguous vs. phonetically ambiguous speech at clear (*p* = 0.0170) and 0 dB (*p* = 0.0011) SNRs, but not at −5 dB (*p* = 0.0915). Across SNRs, ERPs to phonetic tokens were more resilient to noise (Tk1/5; linear contrast of SNR: *t*_70_ = −2.17, *p* = 0.07). In contrast, responses declined systematically for phonetically ambiguous speech sounds (Tk3; *t*_70_ = −2.91, *p* = 0.0098). These neural findings parallel our behavioral results and suggest the categorical (phonetic) representations of speech are more resistant to noise than those that do not carry a clear linguistic-phonetic identity.

### Brain-Behavior Relationships

**FIGURE 6:**
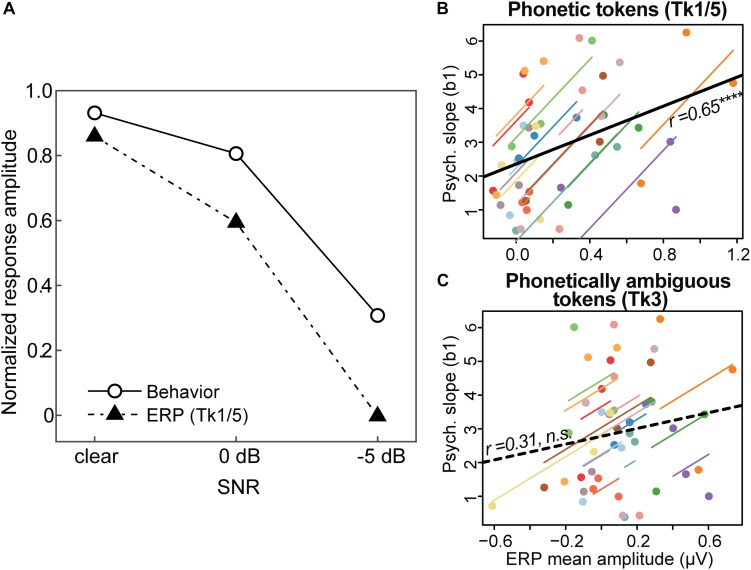
Brain-behavior associations in categorical speech perception. **(A)** Amplitudes of the auditory ERPs (Figure 5B) are overlaid with behavioral data (psychometric slopes; Figure 3B). Neural and behavioral measures are normalized for each participant (Alain et al., 2001), with 1.0 reflecting the largest displacement in ERP amplitude (mean: 180–320 ms; see Figure 5B) and psychometric slopes, respectively. **(B,C)** Repeated measures correlations (rmCorr) (Bakdash and Marusich, 2017) between behavioral CP and neural responses at the single-subject level for **(B)** phonetic (Tk1/5) and **(C)** phonetically ambiguous speech tokens (Tk3). The ordinate measure represents each listeners’ psychometric slope, computed from their entire identification curve (i.e., Figure 3B). Behavioral CP is predicted only by neural activity to phonetic tokens; larger ERP amplitudes elicited by Tk1/5 speech are associated with steeper, more dichotomous CP. Individual lines, single subject fits; thick black lines, overall rmCorr. ****p < 0.0001.

The effects of noise on categorical neural processing closely paralleled the perceptual data. Figure 6A shows the group mean performance on the behavioral identification task and group mean ERP amplitudes (180–320 ms window) to the phonetic speech tokens (Tk1/5). For ease of comparison, both the neural and behavioral measures were normalized for each participant (Alain et al., 2001), with 1.0 reflecting the largest displacement in ERP amplitude and psychometric slopes, respectively. The remarkably similar pattern between brain and behavioral data implies that perceptual identification performance is predicted by the underlying neural representations for speech, as reflected in the ERPs. Indeed, repeated measures correlational analyses revealed a strong association between behavioral responses and ERPs at the single-subject level when elicited by the phonetic (Tk1/5) (Figure 6B; r_*rm*_ = 0.65, *p* < 0.00001, *df* = 29) but not ambiguous (Tk3) tokens (Figure 6C; *r*_*rm*_ = 0.31, *p* = 0.09, *df* = 29). That is, more robust neural activity predicted steeper psychometric functions at the individual level. These findings suggest the neural processing of speech sounds carrying clear phonetic labels predicts more dichotomous categorical decisions at the behavioral level; whereas neural responses to ambiguous (less-categorical) speech tokens do not predict perceptual categorization.

## Discussion

By measuring neuroelectric brain activity during rapid classification of SIN, our results reveal three main findings: (1) speech identification is robust to acoustic interference, degrading only at very severe noise levels (i.e., negative SNRs); (2) the neural encoding of speech is enhanced for sounds carrying a clear phonetic identity compared to phonetically ambiguous tokens; and (3) categorical neural representations are more resistant to external noise than their categorically ambiguous counterparts. Our findings suggest the mere process of categorization—a fundamental operation to all perceptual systems (Goldstone and Hendrickson, 2010)—aids figure-ground aspects of speech perception by fortifying abstract categories from the acoustic signal and making the speech code more resistant to external noise interference.

Behaviorally, we found listeners’ psychometric slopes were steeper when identifying clear compared to noise-degraded speech; identification functions became shallower only at the severe (negative) SNRs when noise levels exceeded that of speech. The resilience in perceptual identification suggests the strength of categorical representations is largely resistant to signal interference. Corroborating our modeling (Figure 1), we found CP was affected only when the input signal was highly impoverished. These data converge with previous studies (Gifford et al., 2014; Helie, 2017; Bidelman et al., 2019) suggesting category-level representations, which are by definition more abstract than their acoustic-sensory counterparts, are largely impervious to surface degradations. Indeed, as demonstrated recently in cochlear implant listeners, the sensory input can be highly impoverished, sparse in spectrotemporal detail, and intrinsically noisy (i.e., delivered electrically to the cochlea) yet still offer robust speech categorization (Han et al., 2016). Collectively, our data suggest that both the mere construction of perceptual objects and the natural discrete binning process of CP help category members “pop out” amidst noise (e.g., Nothdurft, 1991; Perez-Gay et al., 2018) to maintain robust speech perception in noisy environments.

Noise-related decrements in CP (Figure 3A) could reflect a weakening of internalized categories themselves (e.g., fuzzier match between signal and phonetic template) or alternatively, more general effects due to task complexity (e.g., increased cognitive load or listening effort; reduced vigilance). The behavioral data alone cannot tease apart these two interpretations. We can rule out the latter interpretation based on our RT data. The speed of listeners’ perceptual judgments to ambiguous speech tokens (Tk3) were nearly identical across conditions and invariant to noise (Figure 3D). In contrast, RT functions became more categorical (“inverted V” pattern) at more favorable SNRs due entirely to changes in RTs for category members (continuum endpoints). These findings suggest that categories represent local *enhancements* of processing within the normal acoustic space (e.g., Figure 1) which acts to sharpen categorical speech representations. That our data do not reflect gross changes in task vigilance is further supported by two additional findings: (i) lapses in performance did not vary across stimuli which suggests vigilance was maintained across conditions and (ii) ERPs predicted behavioral CP only for speech sounds that carried clear phonetic categories (Figure 6). Indeed, the differential effect of noise on ERPs to category vs. non-category phonemes provides strong evidence that the observed effects reflect modulations in categorical processing. Parsimoniously, we interpret the effects of noise on CP as changes in the relative *sharpness* of the auditory categorical boundary (Livingston et al., 1998; Bidelman et al., 2019). That is, under extreme noise, speech identification is blurred, and the normal warping of the perceptual space is partially linearized, resulting in more continuous speech identification. Stated differently, at high enough levels, noise might challenge speech perception at SNRs where it eliminates differences between clear endpoint and ambiguous tokens in the perceptual space.

It should be noted aforementioned neural effects are probably not soley limited to neural generators in the superior temporal gyrus (i.e., auditory cortex) which generate the majority of the scalp auditory ERP (Picton et al., 1999). There is, for example substantial evidence that perception of ambiguous speech sounds is aided by frontal linguistic brain regions (e.g., inferior frontal gyrus, IFG) (Xie and Myers, 2015; Rogers and Davis, 2017). Similarly, we have shown the differential engagement of IFG vs. auditory cortex during vowel categorization strongly depends on stimulus ambiguity and listeners’ auditory expertise; more ambiguity and less skilled perceivers more strongly recruit IFG (Bidelman and Walker, 2019). Thus, our scalp P2 data most likely reflect an auditory-region-based picture of speech-in-noise categorization. We do not rule out the possibility that complementing information from other brain regions and likely different processing stages that participate over time also aid categorization, especially in noise (Du et al., 2014; Bidelman and Howell, 2016).

On the basis of fMRI, Guenther et al. (2004) posited that the length of time auditory cortical cells remain active after stimulus presentation might be shorter for category prototypes than for other sounds. They further speculated “the brain may be reducing the processing time for category prototypes, rather than reducing the number of cells representing the category prototypes (Guenther et al., 2004, p. 55).” Some caution is warranted when interpreting these results given the sluggishness of the fMRI BOLD signal and inherent difference in the nature of signal that is encoded by ERPs compared to fMRI. Still, our data disagree with Guenther et al. (2004)’s first assertion since ERPs showed larger (enhanced) activations to categorical prototypes within 200 ms. However, our RT data do concur with their second hypothesis. We found RTs were faster for prototypical speech (i.e., RT_*Tk*1/5_ < RT_Tk3_) providing confirmatory evidence that well-formed categories are processed more efficiently by the brain.

Our neuroimaging data revealed enhanced brain activity to phonetic (Tk1/5) relative to perceptually ambiguous (Tk3) speech tokens. This finding indicates categorical-level processing occurs as early as ∼150–200 ms after sound arrives at the ear (Bidelman et al., 2013; Alho et al., 2016; Toscano et al., 2018). Importantly, these results cannot be explained in terms of mere differences in exogenous stimulus properties. On the contrary, endpoint tokens of our continuum were actually the most distinct in terms of their acoustics. Yet, these endpoint (category) stimuli elicited stronger neural activity than midpoint tokens (i.e., Tk1/5 > Tk3), which was not attributable to trivial differences in SNR of the ERPs. These results are broadly consistent with previous ERP studies (Dehaene-Lambertz, 1997; Phillips et al., 2000; Bidelman et al., 2013, 2014; Altmann et al., 2014; Bidelman and Lee, 2015), fMRI data (Binder et al., 2004; Kilian-Hütten et al., 2011), and near-field unit recordings (Steinschneider et al., 2003; Micheyl et al., 2005; Bar-Yosef and Nelken, 2007; Chang et al., 2010), which suggest auditory cortical responses code more than low-level acoustic features and reflect the early formation of auditory-perceptual objects and abstract sound categories.^5^

ERP effects related to CP (Figure 5) were consistent with activity arising from the primary and associative auditory cortices along the Sylvian fissure (Alain et al., 2017; Bidelman and Walker, 2019). The latency of these modulations was comparable to our previous electrophysiological studies on CP (Bidelman et al., 2013; Bidelman and Alain, 2015b; Bidelman and Walker, 2017) and may reflect a modulation of the P2 wave. P2 is associated with speech discrimination (Alain et al., 2010; Ben-David et al., 2011), sound object identification (Leung et al., 2013; Ross et al., 2013), and the earliest formation of categorical speech representations (Bidelman et al., 2013). That the P2 further reflects category access is also supported by the fact ERPs were enhanced to endpoint stimuli and converged with the ambiguous tokens only at the poorest SNR (Figure 5). This latter finding suggests that although endpoint tokens were more resilient to noise than boundary tokens overall, all stimuli probably became perceptually ambiguous in high levels of noise.

Alternatively, P2 differences could reflect increased exposure (or familiarity) effects (Ross and Tremblay, 2009; Ben-David et al., 2011; Tremblay et al., 2014). Under this interpretation, more ambiguous (i.e., less prototypical) sounds near the middle of our continuum would presumably be more unnatural and be less familiar to listeners, which could influence P2 amplitude. Indeed, we have shown listeners’ expertise, and hence familiarity and with sounds in a given domain modulate P2 in speech and music categorization tasks (Bidelman et al., 2014; Bidelman and Lee, 2015; Bidelman and Walker, 2019). In addition, relative P2 amplitude decrease could be associated with phonetic recalibration in the context of hearing the phonetic continuum in different SNRs that may or may not counteract against the noise-induced masking effects. For example, Bidelman et al. (2013) showed that when an ambiguous vowel was classified as [u], P2 amplitude was lower than when the same vowel was perceived as [a]. Thus, a phonetic (re)calibration process might play an important role here in the P2 amplitude differences between end- (Tk1/5) and mid-point (Tk 3) stimuli.

Nevertheless, we found categorical neural enhancements also persisted ∼200 ms after P2, through what appeared to be a P3b-like deflection. Whether this wave reflects a late modulation of P2 or a true P3b response is unclear, the latter of which is typically evoked in oddball-type paradigms. A similar “post-P2” wave (180–320 ms) has been observed during speech categorization tasks (Bidelman et al., 2013; Bidelman and Alain, 2015b), which varied with perceptual (rather) than acoustic classification. This response could represent integration or reconciliation of the input with a phonetic memory template (Bidelman and Alain, 2015b) and/or attentional reorienting during stimulus evaluation (Knight et al., 1989). Similar responses in this time window have also been reported during concurrent sound segregation tasks requiring active perceptual judgments of the number and quality of auditory objects (Alain et al., 2001; Bidelman and Alain, 2015a; Alain et al., 2017). Our findings are also consistent with Toscano et al. (2010), who similarly suggested ERP modulations in the P2 (and P3) time window reflect access to category-level information about phonetic identity. This response might thus reflect controlled processes covering a widely distributed neural network including medial temporal lobe and superior temporal association cortices near parietal lobe (Alain et al., 2001; Dykstra et al., 2016). The posterior scalp distribution of this late deflection is consistent with this interpretation (Figure 4).^6^ Paralleling the dynamics in our neural recordings, studies have shown that perceptual awareness of target signals embedded in noise produces early focal responses between 100–200 ms circumscribed to auditory cortex and posterolateral superior temporal gyrus that is followed by a broad, P3b-like response (starting ∼300 ms) associated with perceived targets (Dykstra et al., 2016). It has been suggested this later response, like the one observed here, is necessary to perceive target SIN or under the demands of higher perceptual load (Lavie et al., 2014; Gutschalk and Dykstra, 2014; Dykstra et al., 2016).

What might be the mechanism for categorical neural enhancements (i.e., ERP_Tk1/5_ > ERP_Tk3_) and their high flexibility in noise? In their experiments on categorical learning, Livingston et al. (1998) suggested that when “category-relevant dimensions are not as distinctive, that is, when the boundary is particularly ‘noisy,’ a mechanism for enhancing separation may be more readily engaged” (p. 742). Phoneme category selectivity is observed early (<150 ms) (Chang et al., 2010; Bidelman et al., 2013; Alho et al., 2016), particularly in left inferior frontal gyrus (pars opercularis) (Alho et al., 2016), but only under active task engagement (Alho et al., 2016; Bidelman and Walker, 2017). While some nascent form of categorical-like processing may occur pre-attentively (Joanisse et al., 2007; Krishnan et al., 2009; Chang et al., 2010; Bizley and Cohen, 2013), it is clear that attention enhances the brain’s ability to form categories (Recanzone et al., 1993; Bidelman et al., 2013; Alho et al., 2016; Bidelman and Walker, 2017). In animal models, perceptual learning leads to an increase in the size of cortical representation and sharpening or tuning of auditory neurons for actively attended (but not passively trained) stimuli (Recanzone et al., 1993). We recently demonstrated visual cues from a talker’s face help sharpen sound categories to provide more robust speech identification in noisy environments (Bidelman et al., 2019). While multisensory integration is one mechanism that can hone internalized speech representations to facilitate CP, our data here suggest that goal-directed attention is another.

The neural basis of CP likely depends on a strong audition-sensory memory interface (DeWitt and Rauschecker, 2012; Bizley and Cohen, 2013; Chevillet et al., 2013; Jiang et al., 2018) rather than cognitive faculties, *per se* (attentional switching and IQ; Kong and Edwards, 2016). Moreover, the degree to which listeners show categorical vs. gradient perception might reflect the strength of phonological processing, which could have ramifications for understanding certain clinical disorders that impair sound-to-meaning mapping (e.g., dyslexia; Werker and Tees, 1987; Joanisse et al., 2000; Calcus et al., 2016). CP deficits might be more prominent in noise (Calcus et al., 2016). Thus, while relations between CP and language-based learning disorders remains equivocal (Noordenbos and Serniclaes, 2015; Hakvoort et al., 2016), we speculate that assessing speech categorization under the taxing demands of noise might offer a more sensitive marker of impairment (e.g., Calcus et al., 2016).

More broadly, the noise-related effects observed here may account for other observations in the CP literature. For example, cross-language comparisons between native and non-native speakers’ CP demonstrate language-dependent enhancements in native listeners in the form of steeper behavioral identification functions (Iverson et al., 2003; Xu et al., 2006; Bidelman and Lee, 2015) and more dichotomous (categorical) neural responses to native speech sounds (Zhang et al., 2011; Bidelman and Lee, 2015). Shallower categorical boundaries for non-native speakers can be parsimoniously described as changes in *intrinsic* noise, which mirror the effects of *extrinsic* noise in the current study. While the noise sources differ (exogenous vs. endogenous), both linearize the psychometric function and render speech identification more continuous. Similarly, the introduction of visual cues of a talker’s face can enhance speech categorization (Massaro and Cohen, 1983; Bidelman et al., 2019). Such effects have been described as a reduction in decision noise due to the mutual reinforcement of speech categories provided by concurrent phoneme-viseme information (Bidelman et al., 2019). Future studies are needed to directly compare the impact of intrinsic vs. extrinsic noise on categorical speech processing. Still, the present study provides a linking hypothesis to test whether deficits (Werker and Tees, 1987; Joanisse et al., 2000; Calcus et al., 2016), experience-dependent plasticity (Xu et al., 2006; Bidelman and Lee, 2015), and effects of extrinsic acoustics on CP (present study) can be described via a common framework.

## Data Availability Statement

The datasets generated for this study are available on request to the corresponding author.

## Ethics Statement

The studies involving human participants were reviewed and approved by the University of Memphis IRB #2370. The participants provided their written informed consent to participate in this study.

## Author Contributions

GB designed the study. LB and AB collected the data. All authors analyzed the data and wrote the manuscript.

## Conflict of Interest

The authors declare that the research was conducted in the absence of any commercial or financial relationships that could be construed as a potential conflict of interest.

## Footnotes

1 Although not central to our questions, some theoretical accounts have described phonetic categorization in terms of a prototype-centric comparator, perceptual magnetic, and statistical learning mechanisms. For review of similar episodic accounts of categorization learning, the reader is referred to Medin and Schaffer (1978) and Neal and Hesketh (1997).

2 Although vowel sounds are perceived less categorically than other speech sounds (e.g., stop-consonants; Pisoni, 1973, 1975; Altmann et al., 2014), they do not carry intrinsic features upon which to make category judgments (formant transitions in consonants, for example, allow comparisons within the stimulus itself) (for discussion, see Xu et al., 2006). In contrast, steady-state features like the F1 contrast of our static vowels lack an intrinsic reference so categorical hearing of these stimuli necessarily requires acoustic features be matched to the best exemplar in long-term memory (Pisoni, 1975; Xu et al., 2006). Thus, we explicitly chose vowels because they are likely to better reflect categorical brain processing as indexed via physiological measures (e.g., Lewis and Bidelman, 2020). Utilizing vowels also ensured the entire stimulus contributed to the categorical percept rather than only the initial transient onset (cf. VOT stimuli) thereby maximizing the possibility that ERPs could be used to differentiate category-level information.

3 While we interpret this contrast to reflect differences in categorial processing, it could also relate to the ambiguity in access to the category label (Hanley and Roberson, 2011; Best and Goldstone, 2019). Still, an ambiguity effect might be expected to produce *larger* ERPs to ambiguous speech (i.e., Tk3 > Tk1/5), which is not the observed direction of our ERP effects (e.g., Figure 5; Tk1/5 > Tk 3).

4 The practical significance of this location effect might be questionable given the shift is less than a half of token. Although speculative, the bias to report/u/under noise could also be related to the phonologically more complex representation for/u/(involving lip rounding, thereby a strong visual cue). Indeed, lip gesture plays a role in the categorical perception sounds (Mottonen and Watkins, 2009).

5 Though we did not establish our endpoint tokens are true global prototypes among the entire acoustic-phonetic space of listeners’ language (cf. perceptual magnet theory; Kuhl, 1991; Guenther and Gjaja, 1996; Iverson and Kuhl, 2000), we contend it is still useful to discuss Tk 1/5 tokens as being “prototypical” because they are the most prototypical among the stimulus set that participants heard.

6 Though there is some evidence that categorization might recruit different neural pathways (e.g., temporal vs. frontal cortex) in skilled vs. unskilled listeners (Bidelman and Walker, 2019), it seems more likely that the speech sounds of our phonetic continuum recruited similar speech-sensitive neural mechanisms which were then modulated by the degree to which categorization mechanisms were successful in sorting them into discrete categories (i.e., changes in P2).
