# Supplementary material for: Effects of Noise on the Behavioral and Neural Categorization of Speech
Source: Front Neurosci. 2020 Feb 27;14:153. doi: 10.3389/fnins.2020.00153 (PMC7057933; doi:10.3389/fnins.2020.00153)
Supplement: Supplementary file 1 [file Data_Sheet_1.DOCX]

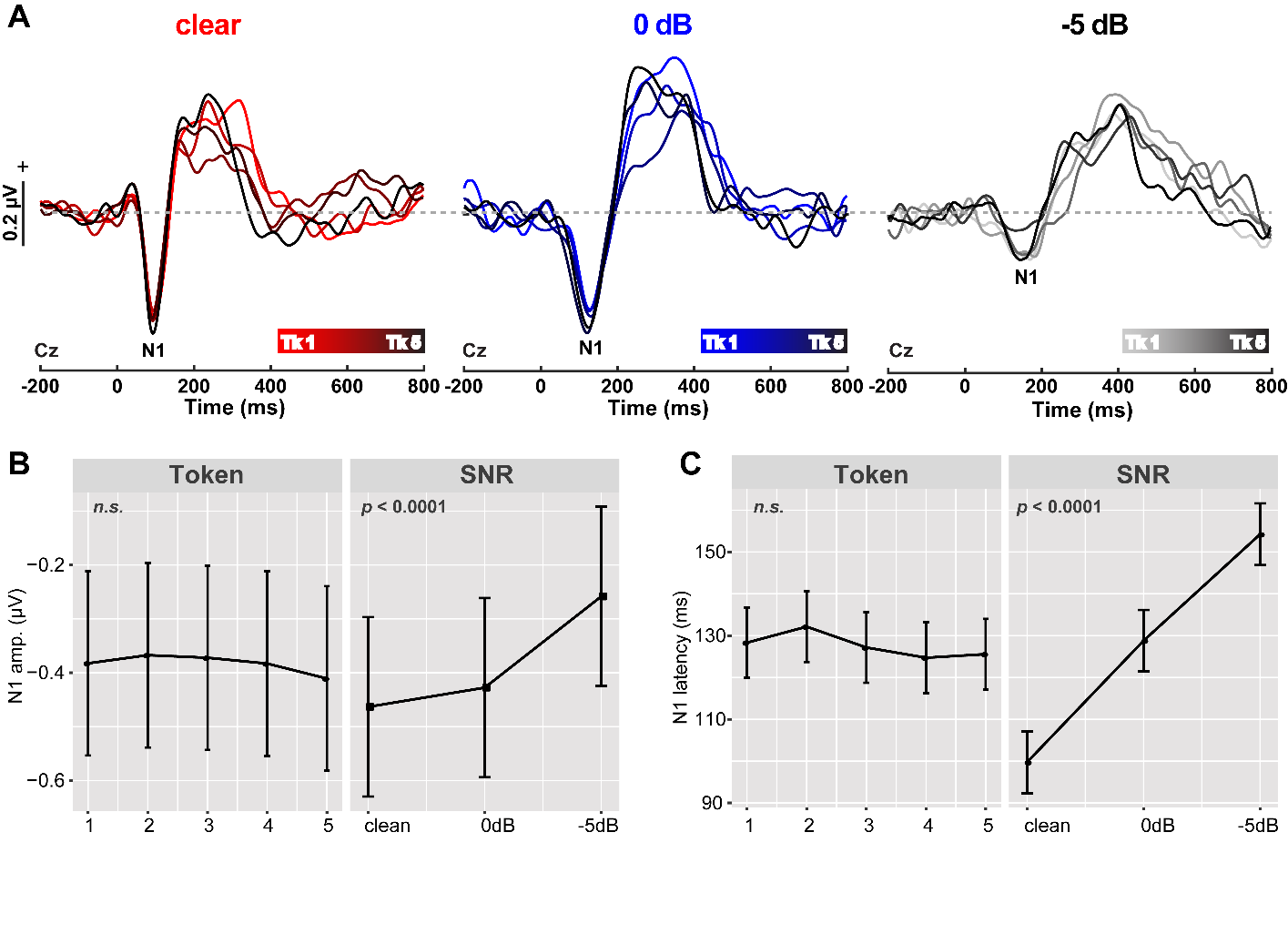


**Fig. S1: (A)** ERPs to the entire speech continuum for clear, 0 dB, and -5 dB SNR speech (Cz electrode). The color gradient denotes the legend (light-to-dark = Tk 1-5). (**B-C**) Marginal means for the token and SNR effect (adjusted for the other factor) for (**B**) N1 amplitude and (**C**) N1 latency. Mixed model ANOVAs showed that N1 response properties were strongly modulated by SNR [N1_amp_: *F_2,196_* = 18.95, *p*<0.0001; N1_lat_: *F_2,196_* = 114.74, *p*<0.0001] but not token number [N1_amp_: *F_4,196_* = 0.27, *p*=0.89; N1_lat_: *F_4,196_* = 0.78, *p*=0.54]. The lack of strong token (categorial) effect on N1 is consistent with previous ERP studies (Bidelman et al. 2013; Toscano et al. 2010) and justifies the examination of the differential P2 response (i.e., Tk1/5 vs. Tk3) reported in the main text. errorbars = 95% CI.

**Behavioral discrimination task**

Customarily, a pairwise (e.g., 1 vs. 2, 2 vs. 3, etc.) discrimination task complements identification functions to establish perceptual equivalence within categories (Pisoni 1973). A discrimination task is somewhat undesirable in the current study given the use of time-varying background noise between stimulus tokens which could artificially inflate discrimination performance if listeners use task-irrelevant cues. For example, differences in stimulus noise between paired comparison tokens could inadvertently provide listeners a cue for making discrimination judgments in 2AFC or ABX tasks. Consequently, our main experiment used identification and RT functions as two behavioral measures of categorization, with the additional advantage that these tasks can also be recorded simultaneously with ERPs. Identification is arguably a truer test of CP since it requires listeners to compare an isolated sound with an internalized memory template to make a categorial judgment. Nevertheless, to further validate our stimuli were heard categorically, we conducted a follow up experiment to measure discrimination performance in an additional sample of N=7 listeners who did not participate in our main ERP experiment (age=24-35 yrs, 6 females; all native English speakers).

We used a 2IFC “same-different” task to measure two-step discrimination (Francis et al. 2003; Pisoni 1973; Xu et al. 2006; Pisoni and Lazarus 1974). Stimuli were pairs of non-adjacent vowels drawn from the continuum (e.g., 1-3, 2-4, 3-5). On each trial, listeners heard a vowel pair that was either identical (e.g., 1-1) or unique (e.g., 1-3) and were asked to respond “same” or “different” as fast and accurately as possible. A total of 110 trials were presented in random order, 60 of which were different trials and 50 of which were same (i.e., catch) trials. The interstimulus interval was set at 500 ms to maximize performance of between- vs. within-category discrimination (Pisoni 1973; Xu et al. 2006). As in the identification task, the next trial commenced after 800-1000 ms (jittered randomly).

Discrimination accuracy was uniformly high (mean= 83%) across token pairs and noise levels (**Fig. S2A**). The superior performance is likely due to the fact listeners can exploit acoustic cues (i.e., continuous surface features of the stimuli) in addition to phonetic (category-level) information in discrimination tasks, particularly for vowels (e.g., Pisoni 1973). A two-way, mixed-model ANOVA conducted on discrimination accuracy (%) revealed main effects of stimulus pair [*F_2,48_* = 6.58, *p*=0.0030] and SNR [*F_2,48_* = 3.53, *p*=0.037], with no interaction [*F_4,48_* = 0.76, *p*=0.55]. Follow-up Tukey-Kramer contrasts revealed peaked discrimination in the -5 dB SNR condition (*t*_48_ = 2.61, *­p*=0.0358) indicative of categorical perception (i.e., heightened discrimination sensitivity at the perceptual boundary). Clear and 0 dB SNR speech discrimination were at ceiling across the board (*p*s> 0.33). Relatedly, the speed of listeners’ discrimination varied solely with noise SNR [*F_2,30_* = 4.81, *p*=0.0155] but did not differ between same and different trial types [*F_1,30_* = 1.05, *p*=0.31] (**Fig. S2B**). Paired contrasts revealed the SNR effect was due to faster RTs in the 0 vs. -5 dB SNR condition.

Collectively, although our vowel stimuli are probably perceived less categorically than other speech sounds (cf. Pisoni 1973; Fry et al. 1962; Altmann et al. 2014), these data support the notion that they are nevertheless binned into discrete perceptual units. Moreover, the fact the most “categorical discrimination” is observed in the most difficult noise condition (-5 dB SNR) further supports the notion that categorization is a mechanism that can aid figure-ground speech perception.


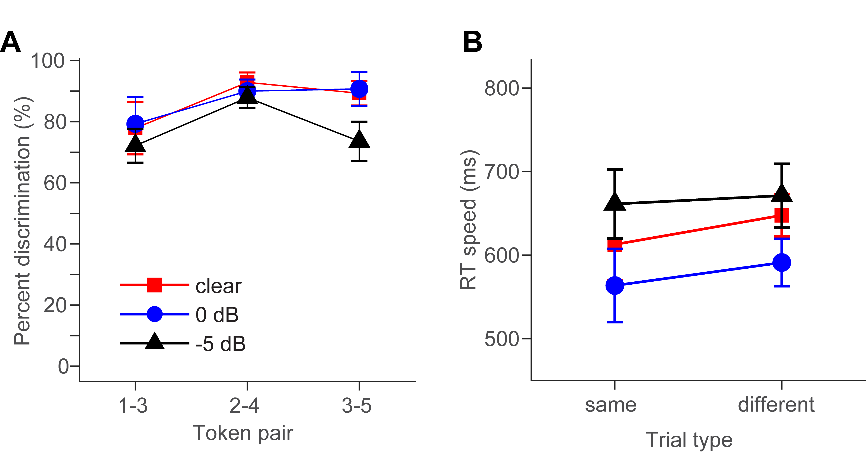


**Fig. S2:** Behavioral discrimination data (n=7). **(A)** Two-step discrimination accuracy. (**B**) Reaction times for discriminating trials with either same vs. different vowel pairs. errorbars = ±1 s.e.m.

**References**

Altmann CF, Uesaki M, Ono K, Matsuhashi M, Mima T, Fukuyama H (2014) Categorical speech perception during active discrimination of consonants and vowels. Neuropsychologia 64C:13-23.

Bidelman GM, Moreno S, Alain C (2013) Tracing the emergence of categorical speech perception in the human auditory system. Neuroimage 79 (1):201-212.

Francis AL, Ciocca V, Ng BK (2003) On the (non)categorical perception of lexical tones. Percept Psychophys 65 (7):1029-1044.

Fry DB, Abramson AS, Eimas PD, Liberman AM (1962) The identification and discrimination of synthetic vowels. Lang Speech 5:171-189.

Pisoni DB (1973) Auditory and phonetic memory codes in the discrimination of consonants and vowels. Percept Psychophys 13 (2):253-260.

Pisoni DB, Lazarus JH (1974) Categorical and noncategorical modes of speech perception along the voicing continuum. J Acoust Soc Am 55 (2):328-333.

Toscano JC, McMurray B, Dennhardt J, Luck SJ (2010) Continuous perception and graded categorization: electrophysiological evidence for a linear relationship between the acoustic signal and perceptual encoding of speech. Psych Sci 21 (10):1532-1540.

Xu Y, Gandour JT, Francis A (2006) Effects of language experience and stimulus complexity on the categorical perception of pitch direction. J Acoust Soc Am 120 (2):1063-1074.
